# Status of short-chain chlorinated paraffins in matrices and research gap priorities in Africa: a review

**DOI:** 10.1007/s11356-021-15924-w

**Published:** 2021-09-03

**Authors:** Vhodaho Nevondo, Okechukwu Jonathan Okonkwo

**Affiliations:** Department of Environmental, Water and Earth Sciences, Faculty of Science, Tshwane University of Technology, 175 Nelson Mandela Drive, Pretoria Central, 0001 South Africa

**Keywords:** POPs, CPs, SCCPs, Matrices, Africa

## Abstract

Chlorinated paraffins (CPs) have been applied as additives in a wide range of consumer products, including polyvinyl chloride (PVC) products, mining conveyor belts, paints, sealants, adhesives and as flame retardants. Consequently, CPs have been found in many matrices. Of all the CP groups, short-chain chlorinated paraffins (SCCPs) have raised an alarming concern globally due to their toxicity, persistence and long-range transportation in the environment. As a result, SCCPs were listed in the Stockholm Convention on Persistent Organic Pollutants (POPs) in May 2017. Additionally, a limit for the presence of SCCPs in other CP mixtures was set at 1% by weight. CPs can be released into the environment throughout their life cycle; therefore, it becomes crucial to assess their effects in different matrices. Although about 199 studies on SCCP concentration in different matrices have been published in other continents; however, there are scarce/or limited studies on SCCP concentration in Africa, particularly on consumer products, landfill leachates and sediment samples. So far, published studies on SCCP concentration in the continent include SCCPs in egg samples, e-waste recycling area and indoor dust in Ghana and South Africa, despite absence of any production of SCCPs in Africa. However, there still remains a huge research gap in the continent of Africa on SCCPs. Consequently, there is a need to develop robust SCCP inventories in Africa since the Stockholm Convention has already developed guidance document in this respect. This review, therefore, examines the state of knowledge pertaining to the levels and trends of these contaminants in Africa and further provides research gaps that need to be considered in order to better understand the global scale of the contaminant.

## Introduction

Chlorinated paraffins (CPs), also known as poly-chlorinated n-alkanes (PCAs), are complex halogenated mixtures which comprise various carbon chain lengths and chlorine atoms and are normally produced by the chlorination of n-alkane feed stocks with molecular chlorine under forcing conditions such as ultraviolet light and high temperature (van Mourik et al. 2015; Wang et al. 2018a). CPs are generally hydrophobic and semi-volatile, ranging from colourless to yellowish liquids (Feo et al. 2009). The three subgroups of CPs are divided based on their carbon chain length: short-chain chlorinated paraffins (SCCPs, C_10_–C_13_), middle-chain chlorinated paraffins (MCCPs, C_14_–C_17_) and long-chain chlorinated paraffins (LCCPs, C > 18) (Wang et al. 2018a; Reth and Oehme 2004).

According to Wang (2010), CPs are mainly categorized under high production volume chemicals in comparison with other persistent organic pollutants (POPs) worldwide. The production of CPs have been increasing significantly since their introduction in the early 1930s (Feo et al. 2009). Globally, an estimation of about > 7 million tonnes of CPs have been produced since the 1930s of which > 1 million metric tonnes are produced per year (Wang 2010). Among other countries, China is listed as the major producer of CPs with recent annual production rates of about 1.05 million tonnes per year (van Mourik et al. 2016). Although not much information is given about the production of SCCPs in Africa, according to a report published in 2015, approximately 10,000 tonnes of CPs were produced per year in South Africa (ICIS 1995). In addition, a study by Babayemi et al. (2019) indicated that large volumes of PVC and other polymers with additives that can contain CPs are imported in huge quantities to African countries with major imports to Egypt, Nigeria, South Africa, Algeria, Morocco and Tunisia, e.g. under Harmonized System (HS) codes HS3901 to HS3907 and HS3917 to HS3926. In total, 33 African countries with available HS data imported approximately 86.14 Mt of polymers in primary form and 31.5 Mt of plastic products between 1990 and 2017 (Babayemi et al. 2019).

Because of their physico-chemical properties such as their varying carbon chain lengths and chlorine percentages, high chemical stability, flame retardance, viscosity, low vapour pressure and strength at low temperature, CPs are added during the production of different consumer products. The consumer products include coolants and lubricants used in metal working fluids, as flame retardants, in polymers mainly polyvinyl chloride (PVC) products, underground mining conveyor belts, paints, sealants and adhesives (Lassen et al. 2014; Tomy et al. 1998). SCCPs can be released into the environment throughout their life cycle, during production, use in industrial processes or production of consumer products and disposal of CP-containing products (Guida et al. 2020). Bidleman et al. (2010) also stated that sources of CPs in the environment can be classified as evaporation and combustion that include volatilization from old in-use or disposed products that contain CPs and polychlorinated biphenyls (PCBs), released during combustion of the consumer products and evaporation from contaminated soils.

Among other CP compounds, SCCPs have triggered a worldwide environmental pollution concern considering their toxicity, persistence, bioaccumulation and long-range transportation in the environment (POPRC 2015). SCCPs were listed under Annex A of Stockholm Convention on POPs in May 2017 (POPRC 2017; UNEP 2017). As decided during the eighth Conference of the Parties (COP-8), SCCPs with chain lengths ranging from C_10_ to C_13_ and a chlorine content greater than 48% by weight should be eliminated (UNEP 2017). Additionally, the content of SCCPs in other CP mixtures was limited to 1% by weight, meaning that CPs with a SCCP content ≥1% are now also considered POPs (UNEP 2019a, b, c). The toxicological effects of SCCPs are particularly highlighted in comparison with those of MCCPs and LCCPs (Wei et al. 2016). SCCPs were documented in the risk profile document of the Persistent Organic Pollutant Review Committee (POPRC) as compounds that may lead to significant health threats in human and animals (POPRC 2015; Wei et al. 2016). Routes of exposure to SCCPs in human include dust inhalation and dietary intake (Fridén et al. 2011). The European Chemicals Bureau (ECB) and the International Agency on Research of Cancer (IARC) have listed SCCPs as carcinogenic compounds (ECB 2007; IARC 1990). Carcinogenicity studies on exposure to CPs in animals have shown that female and male mouse treated for 103 weeks by gavage with 0.125 or 250 mg/kg bw of commercial-grade CP product dissolved in corn oil were dead at 112−114 weeks of age (IARC 1990). Wang et al. (2019b) have associated the toxicology of SCCPs with lethality, hepatotoxicity, developmental toxicity, carcinogenicity, endocrine- and metabolism-disrupting effects and the immunomodulatory effect both in animals and human.

Studies on POPs such as brominated flame retardants (BFRs), polybrominated diphenyl ethers (PBDEs), polychlorinated biphenyls (PCBs) organochlorine pesticides (OCPs) and hexabromocyclododecanes (HBCDs) in different matrices have been carried out in Africa (Asante et al. 2011; Babayemi et al. 2015; Odusanya et al. 2009; Oloruntoba et al. 2019; Sindiku et al. 2015; Mansour 2009; Nkabinde et al. 2018; Adu-Kumi-Jonathan et al. 2019; Vaccher et al. 2020; Katima et al. 2017; Sibiya et al. 2019). However, there is limited information on CP studies in different matrices, with the exception of the studies by Adu-Kumi-Jonathan et al. (2019), Brits et al. (2020) and Möckel et al. (2020). The scarcity of information on SCCPs in the continent can be attributed to lack of analytical capacity. Lack of chemical management, regulations and database of chemicals used could also be among other reasons. However, different studies from different continents have shown the occurrence and concentrations of SCCPs in almost all matrices such as air, water, wastewater, soil, sediment, biota, human blood and breast milk (van Mourik et al. 2016; Li et al. 2017a; Xia et al. 2017a). Additionally, considering that Africa is second to Asia in terms of size and population with increasing industrialization, population and urbanization and prone to legal and illegal imports of goods (Gioia et al. 2014), it, therefore, becomes an important study area for SCCPs. This review, therefore, examines the state of knowledge pertaining to the levels and trends of these contaminants in Africa and further provides research gaps that need to be considered in order to better understand these pollutants on a global scale. Additionally, Africa needs to assess the presence, use and life cycle of SCCPs in order to develop and update its implementation plans to control the use of POPs such as SCCPs.

## SCCP production, industrial uses and environmental releases

### Production

SCCPs, including other CP groups, have been produced commercially in different countries since the 1930s with some minor production already before the 1920s (Glüge et al. 2016). The global production of CPs was estimated to be around 20 and 165,000 tonnes per year, with the United States of America (USA) leading this production (Glüge et al. 2016; Guida et al. 2020). However, between 2006 and 2013, the global production of CPs reached alarming volumes when China took the lead, increasing its production to 260,000 tonnes per year in 2006 and 1,000,000 tonnes per year in 2013 (Guida et al. 2020; Xu et al. 2014). According to Zeng et al. (2012), more than 150 manufacturing factories of CPs are scattered throughout China, which contributes to the widespread occurrence of CPs in China. The overall production of CPs considerably increased at the end of the 1970s, when Japan and European countries began or increased production (IARC 1990; Guida et al. 2020). Wei et al. (2016) reported that SCCPs have been used as replacements for the use of PCBs in Japan; however, the production volume of SCCPs decreased to approximately 500 tonnes per annum in 2002 because of their production being regulated. Fiedler (2010) reported that countries such as South Korea have no record of SCCP production, but an estimation of about 156 tonnes was reported in 2002. India was estimated to have an annual volume production of SCCPs of about 0.11 million tonnes in 2008 (Chaemfa et al. 2014) and currently the second largest producer of CPs. Moreover, the production of SCCPs have ceased in the US, Japan, Canada and Europe. However, van Mourik et al. (2016) reported that MCCPs and LCCPs were still produced as alternatives resulting in the increase of CP production volume per year. The Stockholm Convention has also pointed out that CPs of various chain lengths were produced in 10 other countries (Australia, Brazil, France, Italy, Japan, Russia, Spain, Slovakia, South Africa and England) (UNEP 2019a, b, c). In Africa, according to a report published in 2015, approximately 10,000 tonnes of CPs were produced per year in South Africa (ICIS 1995). In addition to the provided information on the production of SCCPs, Table 1 shows information on countries producing SCCPs or other CPs.

**Table 1:** Countries producing SCCPs or other CPs

Status of SCCP or CP production	Countries
Production of SCCPs between 1993 and 2009	USA, Brazil, Germany, France, the UK, Italy, Japan
Stopped production of SCCPs	Brazil, EU countries, Japan, Russia, United States (US)
Current production of CPs of various lengths	Australia, Brazil, China, France, India, Italy, Japan, Russia, Spain, Slovakia, South Africa, UK
Never had SCCP production	South Africa

Data were compiled from draft guidance on preparing inventories of SCCPs (UNEP 2019a)

### Industrial uses

According to the draft guidance on preparing SCCP inventories, uses of SCCPs have varied between countries over time depending on the need of products and the regulatory frame (UNEP 2019a). SCCPs were mainly used in polyvinylchloride (PVC) as secondary plasticizers, in rubber as flame retardants, metal-working fluids and other lubricants, paints, coatings, sealants, adhesives, textiles and leather and other plastics, such as ethylene-vinyl acetate (EVA), which also includes a range of polymeric products associated with frequent contact or indoor releases (UNEP 2019a). Studies have shown that CP mixtures, mainly SCCPs and MCCPs, are commonly found in consumer products (Gallistl et al. 2017; Brandsma et al. 2019; Yuan et al. 2017a, b; Wang et al. 2018a, b, 2019a, b, c, d, e; Xu et al. 2019a, b, c). As a result, the European Union (EU) has established a regulatory limit of 1500 mg kg^−1^ for SCCPs in consumer products and 1% in other CP formulations. However, after screening of consumer product market in the EU and Norway from 2013 to 2017, a report to the Rapid Exchange of Information System of the European Union (RAPEX) revealed that a wide range of consumer products exceeded the limit of 1500 mg kg^−1^ (Guida et al. 2020). Furthermore, in 2013, the use of SCCPs was prohibited in the US, while the EU restricted their use in 2011. Nevertheless, SCCPs were still applied as flame retardants in conveyor belts in the mining industry and in dam sealants (ESWI 2011). Fiedler (2010) also reported that SCCPs were still applied in different consumer products at rates of about 100 ktonnes per year worldwide.

In Europe, SCCPs are mainly applied in rubber industry, followed by sealants and adhesives, paints and paint coatings and varnishes (ESWI 2011). It was reported that the consumption of SCCPs in these consumer products have increased from 638 to 1254 tonnes per year in 1998 and 713–796 tonnes per year in 2011 (ESWI 2011). In addition, SCCPs were used to replace PCBs and polychlorinated naphthalenes (PCNs) in a wide range of open applications (cables, sealants, adhesives, textiles and leather) (Howard et al. 1975), but with certain exemptions under the Stockholm Convention (Table 2). The Stockholm Convention exemptions included additives in the production of transmission belts in the natural and synthetic rubber industry, spare parts of rubber conveyor belts in the mining and forestry industries, leather industry, in particular fat-liquoring in leather, lubricant additives and for automobile engines. Other exemptions include electrical generators and wind power facilities; oil and gas exploration, petroleum refinery to produce diesel oil and tubes for outdoor decorating bulbs; waterproofing and flame-retardant paints; and adhesives, metal processing and secondary plasticizers in flexible PVC, except in children’s toys (UNEP 2019a; Guida et al. 2020). With the already mentioned information on the production of CPs and the import of polymers in Africa, the occurrence of CPs in different applications becomes quite clear, and the need to also develop robust SCCP inventories and regulations in Africa becomes obvious.

**Table 2:** SCCP content limits reported for their main uses.

SCCP applications	SCCP content in mg/kg
Lubricant	Up to 700,000 (70% weight)
Metal-working fluid	Up to 700,000 (70% weight)
Adhesive/sealant	Up to 300,000 (30% weight)
Paint	Up to 200,000 (20% weight)
Leather	Up to 200,000 (20% weight)
Rubber	Up to 170,000 (17% weight)
Textile	Up to 150,000 (15% weight)
Polyvinylchloride (PVC)	Up to 100,000 (10% weight)
Ethylene-vinyl acetate (EVA) foam	Up to 70,000 (7% weight)

Adapted from UNEP (2019a); Guida et al. (2020)

### Environmental releases

The release of SCCPs into the environment normally occurs anthropogenically during production, industrial use and disposal of SCCP-containing consumer products (Guida et al. 2020). It is proposed that SCCPs are more likely to migrate through vapour phase, while MCCPs and LCCPs are likely adsorbed to particles like dust (Zhou et al. 2018; Brits et al. 2020). Few estimates are available for the releases and bioavailability of CPs from polymeric material lost during product service life due to evaporation or as particles during wear and abrasion of flooring, rubber products, sealants or PVC (Guida et al. 2020). However, a study from the European Commission assumed that about 8% of the SCCPs in sealants are emitted during the product lifetime which is higher than the release rate of PCBs from sealants by evaporation, approximately 0.06% per year (ESWI 2011; Weber et al. 2018). Lassen et al. (2014) reported that wastes consisting of rubber, sealants, adhesives, paints and textiles are a potential source of SCCPs entering into the environment.

While underground mining conveyor belts are considered major sources of SCCPs released into the waste stream (55%) in Europe, followed by sealants (19.8%) and paints (19.8%) (ESWI 2011), it is noteworthy to consider that Africa is endowed with large reserves of mineral resources, which contributes a significant part of the economy (Campbell 2009). Since the beginning of the 1980s, the International Financial Institutions (IFIs) and developing countries have assigned a high priority to the development of mining sector to meet the goal of improving national economic conditions and ultimately reduce poverty. However, faced with the environmental and social impacts of the mining industry, these objectives have been constantly challenged (Campbell 2009). In view of this, it is, therefore, important to mention that underground mining conveyor belts are used in large quantities in the African mining industry, which contributes to the release of SCCPs from this source.

Xu et al. (2014) reported an estimation of about 503 and 1,290 tonnes emission of SCCPs into the atmosphere and water respectively from CP production and application sites in China. CPs in some applications, such as lubricants and metal-working fluids, are largely released to the environment during use (Guida et al. 2020). The usage of metal cutting fluids is listed as one of the largest sources of emission into the atmosphere (246–412 t year^−1^), followed by the synthesis of PVC plasticizers (40.0–58.7 t/year) (Xu et al. 2014). SCCPs released to the environment may also occur during the use of gear oil packaging, fluids used in hard rock mining, fluids and equipment used in oil and gas exploration, manufacture of seamless pipe, metal working and operation of turbines on ships (CPIA 2002). Although a handbook prepared by the Chlorinated Paraffins Industry Association (CPIA) to promote the environmentally safe management of used oils is available, a major challenge is that waste oils are often recovered as fuel and the input of CPs to such fuels increases the risk of corrosion (PDEP 2019). Also, oils with a halogen content above 1000 mg kg^−1^ might be considered hazardous waste in some countries (PDEP 2019) or might need destruction in a hazardous waste incinerator if the halogen organic substance content is above 10,000 mg kg^−1^ (European Commission 2010).

Landfills are identified as a potential source of SCCPs released into the environment due to the occurrence of SCCP-containing waste in landfills (Lassen et al. 2014). Release pathway of SCCPs from landfills includes leaching, run-off and volatilization (POPRC 2015; Fiedler 2010; Feo et al. 2009; Zeng et al. 2013b). According to Weber et al. (2011), POPs are released from landfills in leachates at varying degrees depending on their physical-chemical properties. SCCPs and other CPs are detected in landfill leachates at levels up to 614 μg L^−1^ (Weber et al. 2011). Considering the higher historical production of CPs compared to other POPs, the amount of waste containing SCCPs and other CPs and the quantity CPs released to the atmosphere is also expected to be high (Glüge et al. 2016). Reports indicated that since the use of CPs started in the 1930s, it can be assumed that a large amount of waste containing SCCPs may have been already disposed into landfills and dump sites since hazardous waste management capacity and practices were not fully developed until the 1970s (UNEP 2018; ESWI 2011). Furthermore, in developing continents, particularly in Africa, polymeric products are disposed either by landfilling or open dumping (Sibiya et al. 2017). Studies by Babayemi et al. (2015, 2018) elaborated that polymeric wastes are disposed with municipal solid waste (MSW) with minimal formal recycling of approximately 10%. It was further explained that there is hardly any thermal or energy recovery from waste (incinerators or cement kilns) in Africa. Thus, the by far largest share of polymeric waste ends up in dump sites or landfills (Babayemi et al. 2019).

Open burning of solid waste is also a common practice in most African countries inclusive of polymeric products (Babayemi et al. 2014). As a result of lack of effective solid waste management, mixed wastes including polymers end up in dump sites where they are burnt, resulting in the release of POPs into the environment (Oloruntoba et al. 2019). Studies have further shown that depending on the type and use, polymeric products contain a wide range of additives such as plasticizers, flame retardants, antioxidants, acid scavengers, light and heat stabilizers, lubricants, pigments, antistatic agents, slip compounds and thermal stabilizers which are used for various purposes (Geyer et al. 2017; GIA 2008; Rajaram 2009; Hahladakis et al. 2018; UNEP 2019b). Many of these additives are known to have toxic effects, and some are classified as endocrine disrupting chemicals. Several others have been listed as POPs, including PBDEs, hexabromobiphenyls (HBB), HBCD, SCCPs and the fluorinated tensides like perfluorooctanoic acid (PFOA) (Babayemi et al. 2019). These releases can therefore be regarded as sources of SCCPs and other CPs to the air, soils, industrial areas and in house dust resulting in human exposure (Fridén et al. 2011; Hilger et al. 2013; Lucattini et al. 2018).

## Routes of exposure and toxicity

### Routes of exposure

Considering the use of SCCPs as additives in diverse consumer applications, continuous human exposure is likely to occur from this route. The major routes of exposure to SCCPs in human include air inhalation, dust ingestion and dermal absorption (EFSA 2020). Despite the scarcity of general information on human exposure to SCCPs, it is reported that ingestion of contaminated food could be a major intake pathway (Liu et al. 2020a, b). In a study conducted by Fridén (2010), dietary intake accounts for about 85% of non-occupational exposure to SCCPs in Sweden, while the same conclusions were drawn from the studies that were undertaken in Canada and China (Gao et al. 2018). Yuan et al. (2017a, b) conducted a study on CP leaching from kitchen food blenders in the European market and reported that about 75% of the hand-held food blenders tested released elevated levels of CP mixtures of all chain lengths (C_6_–C_22_) which ranged from 0.10 to 120 μg with potential human exposure. Since all CP mixtures detected had a SCCP content significantly above 1% (4–59%; average of 28%), the used mixtures would also be classified as POPs (Guida et al. 2020). Various amounts of CPs were also detected in baking ovens from German kitchens (Gallistl et al. 2018). MCCP levels of up to 1000,000 mg g^−1^ were measured inside baking oven in 10 out of 21 samples (48%), and in seven of these, oven SCCP content was below the limit of quantification meeting the European Union and Stockholm Convention requirement of a SCCP content of less than 1% in MCCP. However, in 3 cases, the SCCP content was 2.8%, 5.0% and 14.4% of the total CP content (Gallistl et al. 2018). Other studies by Wang et al. (2018a, b, c) measured CP content in several consumer products with direct contact to human food (disposable tableware, beverage bottles, nursing bottles and lunch boxes) and also performed leaching test. Measured SCCP concentration ranged from 0.02 to 69 mg kg^−1^, while MCCP concentration ranged from 0.02 to 69 mg kg^−1^. Furthermore, to investigate the migration of CPs from food packaging into food, food simulants (water, 3%; acetic acid, 15%; ethanol and hexane) were used, and an average migration efficiency of 12% and 1.5% for SCCPs and MCCPs were reported, respectively. SCCPs (C_10_) and lower chlorinated (C_6_ and C_7_) showed the highest migration. With these findings, it is important to highlight that since there is lack of CP regulations on uses in most African countries, SCCP oils might enter sensitive uses like lubricants in food production sector leading to a larger population exposed to their toxicological effects.

### Toxicity

The toxicity of SCCPs has raised an alarming public concern globally leading to a number of research studies reviewed by Wang et al. (2019b). According to Dong et al. (2020), both plants and animals are susceptible to the toxic effects of SCCPs because of their bioaccumulation in the environment. A report from the European Food Safety Authority (EFSA) have explained that the detection of CPs in human blood and milk samples indicate that CPs are absorbed to some extent in humans and the detection of CPs in umbilical cord blood indicates that CPs can be transferred to the foetus (EFSA 2020). The toxicology of SCCPs have been associated with lethality, hepatotoxicity, developmental toxicity, carcinogenicity, endocrine- and metabolism-disrupting effects and the immunomodulatory effect both in animals and humans (Wang et al. 2019b; EFSA 2020). In a study conducted by Ren et al. (2018) on rats and mice administered with SCCPs for 2 years, survival rates of female mice were at 250 mg kg^−1^ day^−1^ after the 100th week, and that of rats at 312 mg kg^−1^ day^−1^ after the 90th week, which were significantly less than those of respective controls. Another study by Wang et al. (2019d) demonstrated that exposure of male C57Bl/6 mice to 100 mg kg^−1^ day^−1^ SCCPs (52% chlorination, vehicle corn oil) for 28 days caused immune cell infiltration in the liver, which indicated a potential of liver damage although the liver weight was not affected. The International Agency for Research on Cancer (IARC 1990) reported that C_10_-SCCPs (60% chlorination) were found to be carcinogenic to experimental animals and possibly carcinogenic to humans. Wang et al. (2019d) also demonstrated that mice exposed to C_9-13_-CPs (vehicle corn oil) altered the expression of cancer-related genes, implying that these cancer-related genes may be involved in SCCP-induced carcinogenicity. Furthermore, SCCPs are known to be endocrine disruptors. Treatment of male rats with chlorowax 500C and cerclor 56 L at 1 g kg^−1^ day^−1^ (vehicle corn oil) by gavage for 14 days significantly reduced the levels of plasma thyroid hormone (TH), 32% and 39% lower for free thyroxine (FT4) and 26% and 35% lower for total T4 (TT4), respectively (Wyatt et al. 1993). However, in contrast, these two SCCPs significantly caused 143% and 117% increase in plasma thyroid stimulating hormone (TSH) levels, respectively (Wyatt et al. 1993). With consistent observation of those previous studies, Gong et al. (2018) reported that male rats exposed to 100 mg kg^−1^ day^−1^ SCCPs (vehicle corn oil) for 28 days showed decreased levels of plasma FT4, free triiodothyronine (FT3) and hepatic T4 and increased levels of plasma TSH and hepatic T3.

## SCCP studies in different continents

Different studies have indicated the ubiquity of SCCPs in diverse environments and matrices, including industrialized regions as well as remote areas (Bayen et al. 2006; Wei et al. 2016; Zeng et al. 2013a). SCCPs have been detected in matrices including air, water, sewage sludge, sediment, soil and biological tissues (Diefenbacher et al. 2015; Chang et al. 2016; Brandsma et al. 2017; Muscalu et al. 2017; Pellizzato et al. 2009), with majority of studies being conducted in China and Germany (van Mourik et al. 2015). While data is also emerging from other regions, to our knowledge, only the existing studies on the concentrations of SCCPs in different matrices are discussed below:

### Atmosphere

Niu et al. (2020) analysed atmospheric SCCP concentration in the Yangtze River Delta in summer (June–September 2011) and winter (November 2011–February 2012) which ranged from 2.27 to 23.6 μg g^−1^ and 3.90 to 26.2 μg g^−1^, respectively. The determined SCCP concentrations were comparable with those obtained in summer and winter in other studies in China (Wang et al. 2019c; Wang et al. 2013; Li et al. 2018). Additionally, the obtained concentrations were also higher than those obtained in Japan and South Korea (Li et al. 2012), India and Pakistan (Chaemfa et al. 2014) and Switzerland (Diefenbacher et al. 2015). Another recent study by Liu et al. (2020a) analysed SCCP concentration in PM_2.5_ samples from Chinese cities, and ∑SCCP concentrations in 10 cities were 19.9 ± 41.1 ng (1.98–274 ng m^−3^). Compared with previous atmospheric SCCP concentration, only Huang et al. (2017) and Li et al. (2019) reported SCCP concentration in PM_2.5_ samples. Globally, the obtained results were generally higher than those in Japan, South Korea, India, UK and Norway (Borgen et al. 2002; Barber et al. 2005; Li et al. 2012; Chaemfa et al. 2014). Wang et al. (2018b) also determined SCCP concentration in air samples from farmlands and villages surrounding the CP production plant in China. SCCP concentration in gas phase and particle phase ranged from 82 to 316 ng m^−3^ and 7 to 17 ng m^−3^, respectively, and ∑SCCP concentrations in air samples were lower as the distance from the CP plant increased. In Australia, van Mourik et al. (2020b) determined SCCP concentration in ambient air samples from urban (UR), rural (RU) and remote areas (RE). SCCP concentrations in the gas phase from samplers deployed at two remote sites (RE_1-2_) were below MDL and if ∑SCCP concentration above MDL, ranged from 11 (RU_5_) to 170 (RU_1_) ng/sampler with ∑SCCP concentration of 61 ng/sampler. The ∑SCCP concentrations were 28-fold higher than those of PCBs measured with the same sampler at the same site in 2012 (Wang et al. 2015).

### Indoor dust

Brits et al. (2020) analysed short-, medium- and long-chain chlorinated paraffins in indoor dust in South Africa. ∑SCCP concentrations in freshly collected dust samples were 17 μg g^−1^ and 14 μg g^−1^ in dust samples collected from household vacuum cleaner bags. The obtained concentrations were higher than those from studies conducted in Australia, Canada, Germany and Sweden (He et al. 2019; Shang et al. 2019; Hilger et al. 2013; Wong et al. 2017) and lower than those from Australia, China and the UK (Wong et al. 2017; Chen et al. 2018; Liu et al. 2017; Shi et al. 2017). Chen et al. (2016) developed a method for extracting SCCPs from indoor dust samples in Taiwan. In application of the method, SCCP concentrations were detected in all indoor dust samples with concentrations ranging from 1.2 to 31.2 μg g^−1^. The determined concentrations were similar with those reported by Fridén et al. (2011) and Hilger et al. (2013).

### Aquatic environment

According to Houde et al. (2008), SCCPs released during production and use have entered water bodies and have been transferred to sediment and aquatic organisms resulting in bioaccumulation through the food chain. Globally, bioaccumulation and trophic magnification of SCCPs have been studied (Houde et al. 2008; Li et al. 2017a; Ma et al. 2014b; Sun et al. 2017; Zeng et al. 2017a). In the aquatic environment, SCCPs are more likely to be adsorbed in sediment since they have low water solubility and high carbon partition coefficients (Environment Canada 2008). There are quite a number of studies that have been reported globally (Wang et al. 2019a; Pan et al. 2018; Pan et al. 2020; Huang et al. 2019a; Wang et al. 2020a; Labadie et al. 2019).

#### River water

Pan et al. (2020) conducted a study in April 2014 to determine SCCP concentration in a river–estuary system in China. SCCP concentration ranged from ND to 470 ng/L with ∑SCCP concentration of 43 ng L^−1^, and C_10_ and C_11_ groups were dominant congener groups in the dissolved phase. The obtained highest SCCP concentration of 470 ng L^−1^ was much higher than those obtained from the influent waters of the municipal sewage treatment plants in Beijing and Japan (Wei et al. 2016; Zeng et al. 2013b). Wang et al. (2019a) reported ∑SCCP concentration ranging from 15.0 to 1640 ng L^−1^ in river water samples. The results obtained were comparable to those obtained from other studies in China (Chang et al. 2016) but were also higher than in other studies (Zeng et al. 2011b; Sun et al. 2017; Houde et al. 2008; Coelhan 2010; Gandolfi et al. 2015). Huang et al. (2019a) also reported SCCP concentration in seawater samples in August 2012 from the Pearl River Estuary, South China. SCCP concentration ranged from 180 to 460 ng L^−1^ with ∑SCCP concentration of 270 ± 66 ng L^−1^ which were higher than the concentrations reported by Ma et al. (2014a), but much lower than those reported by Nicholls et al. (2001) while comparable to those reported by Wang et al. (2019a).

#### Sediment

A study was conducted by Wang et al. (2019a) in May 2016 in river water and sediments in Shanghai, China. The ∑SCCP concentrations in sediments were in the range of not detected (ND)–2020 ng g^−1^. The obtained concentrations were lower than those obtained in other studies while similar to the ones obtained in some (Chen et al. 2011; Gao et al. 2012; Sun et al. 2017; Xu et al. 2019b; Zeng et al. 2017b; Qiao et al. 2016), whereas higher in comparison with two studies (Qiao et al. 2017; Iozza et al. 2008). However, the Mann-Whitney *U* test indicated that MCCP concentrations in sediments were higher than those of SCCPs (*p* <0.001), which was attributed to the use pattern of commercial CP formulations in China. Thus, Shanghai aquatic system is mainly contaminated by MCCPs than SCCPs. Another study by Pan et al. (2018) determined SCCP concentration in sediments from industrial, urban and rural areas of the Laizhou Bay, North China, from 14 September 17 to October 2009. SCCP concentration ranged from 8.4 to 2000 ng g^−1^ with ∑SCCP concentration of 160 ng g^−1^ dw. In comparison with other studies, the results were similar to those obtained from previous studies (Pan et al. 2011a; Pan et al. 2011b), which confirmed that emissions from local factories were likely the main source of SCCPs in river sediment. Qiao et al. (2017) also determined SCCP concentration in sediments from 13 locations in the middle reaches of the Yangtze River, which ranged from 4.19 to 41.6 ng g^−1^ dw and chlorine contents ranging from 61.8 to 63.8%. The obtained results were similar to those of Zeng et al. (2012) and Zeng et al. (2013a), but lower than those obtained by Gao et al. (2012) and Ma et al. (2014a). Furthermore, SCCP concentrations were higher in sediment samples collected from industrialized cities where electronics manufacturing, plastic factories and petrochemical plants are located. Factors such as population density and human activities play a major role in the use and disposal of CPs; therefore, the concentrations of SCCPs are expected to be higher in urban areas than in rural areas.

In Central Europe, from July 2003 to January 2004, Přibylová et al. (2006) determined SCCP concentration in selected riverine sediments from 11 rivers (Labe, Bilina, Ohre, Vltava, Jihlava, Dyje, Svitava, Morava, Becva, Mala Becva and Drevnice) of the Czech Republic. Highest SCCP concentration of 89 ng g^−1^ was detected in sediment samples from Ohre River, which was influenced by a local factory where a range of crimping connectors and lugs for aluminium or copper cables are produced. Other SCCP sources to the Ohre River included the chemical factory where water-based dispersion and polymers are produced which is located close to the river. Additionally, other studies have indicated SCCP concentration of up to 19 μg g^−1^ ww and 10 μg g^−1^ in sediment samples from Norway and Japan, respectively (Boreen et al. 2003).

### Soil

Wu et al. (2020) collected soil samples in June 2018 from CP production plant brownfield site in China. ∑SCCP concentrations were detected at ND–5090 ng g^−1^ dw which indicated that CP production caused soil contamination of SCCPs. Only two studies were comparable with the obtained results (Xu et al. 2016; Wang et al. 2018b). The obtained results were also much higher than those reported by Bogdal et al. (2017), Wang et al. (2014) and Zeng et al. (2011a). SCCP concentrations ranging from 37.5 to 995.7 ng g^−1^ dw in soil samples from a chemical industrial park were also determined by Huang et al. (2020), which were lower than the concentrations determined in Spain and Yangtze River Delta (Castells et al. 2008; Chen et al. 2011). Wang et al. (2020a) reported SCCP concentration in soil samples ranging from 79 to 948 ng g^−1^ with ∑SCCP concentration of 348 ng g^−1^. Zhao et al. (2019) also reported ∑SCCP concentration in coastal soil samples from Shandong Peninsula ranging from 50.06 to 266.3 ng g^−1^.

### Fish

Wang et al. (2020b) reported SCCP concentration in coral reef fish from the Nansha Islands, South China Sea. SCCP concentration in fish samples from Zhubi Reef and Yongshu Reef ranged from 37.9 to 20.200 ng/g lipid weight (lw) and 75.7–25.400 ng g^−1^ lw, with ∑SCCP concentration of 3700 ± 6000 ng g^−1^ lw and 5200 ± 7340 ng g^−1^ lw, respectively. Different studies have shown that the lipid content is a crucial factor affecting SCCP bioaccumulation in marine environment (Yuan et al. 2012; Ma et al. 2014b; Zeng et al. 2017a). In contrast, the obtained results were different from those of Pearl River Estuary, where lipid level was not the predominant influencing factor for SCCP concentration in marine organism (Huang et al. 2019). Another study by Huang et al. (2019b) has shown SCCP concentration in fish samples from the Pearl River Delta and Yangtze River Delta. The concentrations of SCCPs in farmed freshwater fish and wild sea fish ranged from 220 to 51000 ng g^−1^ lw and 900 to 7300 ng g^−1^ with ∑SCCP concentration of 5900 ± 8100 ng/g lw and 3000 ± 1600 ng/g lw, respectively. Labadie et al. (2019) determined SCCP concentration in fish samples from the Rhone River basin, France. SCCP concentration ranged from 0.3 to 10.6 ng g^−1^ wet weight (ww) which were in the same order of magnitude with those reported by Saborido Basconcillo et al. (2015), Herzke et al. (2013) and Parera et al. (2013).

### Eggs

In Africa, Adu-Kumi-Jonathan et al. (2019) analysed egg samples from Agbogbloshie and Accra supermarket, which was the first SCCP analysis in eggs from Africa. SCCP concentration ranged from 62 to 2067 ng g^−1^. Compared with SCCP concentration from China, the concentrations were much lower. Zeng et al. (2016) determined SCCP concentration in goose egg samples ranging from ND to 150000 ng g^−1^ lw in South China. Another study by Martínez Prats (2015) analysed SCCP concentration in gull eggs of two species (*Larus michahellis* and *Larus audouinii*) from three natural protected areas in Spain (Delta del Ebro Natural Park; Montgrí, Illes Medes i Baix Ter Natural Park; and Islas Atlánticas de Galicia Natural Park). SCCP concentration determined in egg samples of *Larus michahellis* ranged from 1.78 to 3.70 ng g^−1^ ww, which were notably lower than those that were determined in *Larus audouinii* ranging from 4.40 to 5.08 ng g^−1^ ww. Morales et al. (2012) also conducted a study in gull eggs of two species (*Larus michahellis and Larus audouinii*) from the Ebro delta Natural Park, and SCCP concentrations were detected at 4536 ± 40 pgg^−1^ ww in *Larus michahellis* and 6364 ± 20 pg g^−1^ ww in *Larus audouinii.*

### Consumer products

According to Gallistl et al. (2017), recent studies on SCCPs in consumer products have shown their occurrence and concentrations in different ranges. Guida et al. (2020) also confirmed that the most available data on CPs in consumer products are for SCCPs (Table 3). In China, Wang et al. (2018a) conducted a CP concentration study in domestic polymeric products, and highest SCCP concentration of up to 19% of the product weight was determined in PVC cable sheaths and an average mass fraction of 8.4% in cable sheaths and 0.25% in PVC floorings. SCCP mean concentrations of about 1.3% were also determined in PVC products and rubber samples originating from rubber tracks, tyres and conveyor belts, which exceeded the limit proposed by the Stockholm Convention (Wang et al. 2018a). Furthermore, the Rapid Exchange of Information System of the European Union (RAPEX) determined highest concentration of SCCPs in squeeze toy which is commonly mouthed by children of about 100.00 mg kg^−1^—10% of the product weight (UNEP 2018). Extremely high concentrations of SCCPs which exceeded the European Union and the Stockholm Convention regulating limits were detected in different consumer products such as sport equipment (exercise tube), plastic bath toy, yoga mats, breastfeeding pillow and selfie stick cord of about 90,000 mg kg^−1^, 71.00 mg kg^−1^, 69.00 mg kg^−1^, 60.00 mg kg^−1^ and 45.700 mg kg^−1^, respectively (UNEP 2018). Another study that was conducted on CP leaching from kitchen blenders in the European market reported that 75% of the hand-held food blenders tested released high concentrations of CP mixtures ranging from 0.10 to 120 μg of all chain length (Yuan et al. 2017b).

**Table 3:** SCCP concentrations in consumer products

Consumer product sample	SCCP concentrations (mg kg^−1^)
PVC (12)	78 (30–3500)
Cables and cards (15)	13.611 (1,100–45,700)
Covers and packing (8)	15.737 (2,600–60,000)
Leather (artificial) (13)	3954 (1,100–14,000)
Sports equipment (18)	19.056 (1,800–90,000)
Stickers (5)	11.600 (2,000–18,000)
Toys (20)	26.893 (1,900–100,00)
Domestic products (19)	11.032 (700–47,000)
PVC (21)	40.770 (ND–13,144)
Rubber	614 (ND–13,144)
PET (19)	0.2 (ND–3)
PE (5)	0.1 (0.02–0.3)
PP (18)	4 (ND–69)
Food packaging (20)	2 (0.01–8.3)
Rubber track products (15)	3639 (14–12,800)
Adhesives	3344 (62–7,140)
Rubber granulate (10)	3 (ND–24)
Car tires	1 (ND–2)

*ND* not detected; data were compiled from a table by Guida et al. (2020)

### Landfill sites and open dumping sites

Guida et al. (2020) stated that the landfill environment forms part of a research gap in CP studies in environmental samples that needs to be investigated and assessed. Considering the fact that at the end of their life cycle, SCCP-containing consumer products normally enter the waste stream as part of municipal solid waste (MSW), the landfill environment becomes another important source of release of SCCPs into the environment that should be studied. Li et al. (2020) conducted a study in landfill soils of the Tibetan Plateau, and the concentrations of SCCPs ranged from 56.8 to 1348 ng g^−1^ dw and ∑SCCP concentration at 434 ng g^−1^. In that study, the highest concentration of SCCPs of about 1348 ng g^−1^ was determined from soils inside the Lhasa landfill which was attributed to the inappropriate disposal of SCCP-containing waste. In another study, high concentrations of SCCPs were determined in landfill sediment from different locations in the south of Norway, which ranged from 330 to 19.400 ng g^−1^, which was due to the deposition of waste from mechanical engineering or shipping industries (Borgen et al. 2003). Furthermore, a number of studies conducted on landfills in China have focused on SCCP concentration that are determined in soil and sediment samples of e-waste dismantling areas than in landfill leachate and sediment samples. Different research studies have shown that concentrations of SCCPs in e-waste dismantling areas are significantly higher than in other sampling areas (Kalinowska et al. 2019). Yuan et al. (2017a) determined the highest concentrations of SCCPs in soils of an e-waste dismantling area, and ∑SCCP concentration ranged from 30.4 to 530 ng g^−1^ dw. Xu et al. (2019b) determined concentrations of SCCPs in soil and sediment samples of an e-waste dismantling area as well. SCCP concentration ranged from 68.5 to 2.20 × 10^5^ ng g^−1^ and 32.5 to 1.29 × 10^4^ ng g^−1^, respectively. Concentrations of SCCPs in soil samples were higher than in sediment samples, which were attributed to the distance between sampling points around the e-waste dismantling centres. Concentrations of SCCPs in soil and sediment samples may vary based on factors such as land-use type, proximity to sources and soil profile (Kalinowska et al. 2019).

According to Möckel et al. (2020), it can be assumed that domestic e-waste and industrial wastes are disposed of at ordinary municipal waste sites in developing countries. Möckel et al. (2020) investigated soil pollution at a major e-waste recycling site in West Africa. SCCP concentrations ranging from 69 to 1600 ng g^−1^ and 145 to 28.000 ng g^−1^ were determined in Kingtom and Agbogbloshie e-waste waste sites, respectively. Compared to literature data, the lowest SCCP concentration determined in the Agbogbloshie and Kingtom samples was similar to slightly higher than concentrations determined in ambient soils in China, UK and Norway (Xu et al. 2016; Halse et al. 2015). However, the highest SCCP concentration in Kingtom samples was similar to concentrations reported in farmland soils irrigated with wastewater and soils close to CP production plant (Zeng et al. 2011a), whereas the highest concentrations determined in Agbogbloshie samples were similar to those recently published in China (Xu et al. 2019b). To date, there are no study reports on concentrations of SCCPs in landfill leachates.

### Humans

According to Xia et al. (2017b), there is a concern about the presence of environmental pollutants in human breast milk because the pollutants could negatively affect the health of breastfeeding infants. In pregnant women, SCCPs normally accumulate in their blood and milk and then transmit to their foetus through either placental transfer or breastfeeding transfer (Aamir et al. 2019; Yang et al. 2018; Lyche et al. 2015). Due to their immature metabolism, infants are more vulnerable than older humans to potentially harmful chemicals (Darnerud et al. 2001).

#### Breast milk

Recently, Liu et al. (2020b) reported ∑SCCP concentration of about 30.3 ng g^−1^ ww in breast milk samples from China. Compared with other studies, the concentrations were of the same magnitude with those reported by Yang et al. (2018). Xia et al. (2017a) also determined SCCP concentration in breast milk samples in 2007 and 2011 in rural China. ∑SCCP concentration ranged from 68.0 to 1580 ng g^−1^ lw and 65.6 to 2310 ng g^−1^ lw in 2007 and 2011, respectively. The obtained results varied by about three orders of magnitude. Cao et al. (2017) also determined SCCP concentration in breast milk samples from Beijing which ranged from below the method detection limit, MDL <20–54 ng g^−1^ lw. In the UK, Thomas et al. (2006) determined SCCP concentrations in human breast milk that ranged from <71 to 820 ng g^−1^ lw.

#### Human serum

Liu et al. (2020b) reported ∑SCCP concentration in maternal and placenta human serum samples of about 117.1 ng mL^−1^ and 70.0 ng mL^−1^, respectively, which were in the same range with those reported by Aamir et al. (2019) and Qiao et al. (2018). Ding et al. (2020) also determined SCCP concentration in serum samples of about 107 ng g^−1^ ww, which were comparable with those previously reported in blood samples (Qiao et al. 2018). SCCP concentrations in serum samples ranging from 1.00 to 5.45 ng mL^−1^ were determined by Zhou et al. (2019), which were lower than those obtained in plasma (Li et al. 2017b) and human blood (Xu et al. 2019c). In Australia, a study done by van Mourik et al. (2020a) showed SCCP concentration in human male serum samples which ranged from <MDL to 0.68 with ∑SCCPs of 0.54 ng g^−1^ ww. The concentrations obtained were lower than those obtained in human, maternal and cord serum samples from China (Zhou et al. 2019; Qiao et al. 2018). In addition to the SCCP studies reviewed above, Table 4 summarizes SCCP studies in different matrices from different continents.

**Table 4:** SCCP studies in various matrices

Matrices		Sites and time of sample collection	Concentrations
Atmosphere	Dust	Plastic tracks and basketball courts in Beijing, China, 2015	5429 and 5139 μg/g
	Indoor PM_10_, PM_2.5_ and PM_1.0_ samples	Beijing, China, 2016	38.3–87.7 (mean: 61.1), 16.8–49.4 (mean: 31.4) and 6.4–32.5 (mean: 20.7) ng/m^3^, respectively
	Indoor dust and air	Beijing, China, 2016	82 g/g and 80 ng/m^3^, respectively
	Dust falls	A Chinese building mall and central air conditioner filter dusts from a newly opened Chinese shopping mall in Dalian, China, 2015	6.0 to 361.4 μg/g and 114.7 to 707.0 μg/g, respectively
	Air	Dalian, China, in 2010 and 2016	15.12 to 66.44 (mean: 30.26) ng/m^3^ and 65.30 to 91.00 (mean: 78.15) ng/m^3^, respectively
	Dusts	E-waste recycling workshops, local residential homes, exterior street surfaces and control homes, collected in a mega e-waste recycling industrial park in South China, between 2016 and 2017	246–19,900 (mean: 5600) μg/g, 34.5–2030 (mean: 580) μg/g, 32.4–982 (mean: 501) μg/g and 27.8–173 (mean: 59) μg/g, respectively
	Indoor particles	The Pearl River Delta, China, 2017	3.3 to 43.2 (mean: 13.4) ng/m^3^
	Indoor particles	Harbin, China, in 2013	10.1 to 173.0 (mean: 53.6) μg/g
	Air	Inside and outside one CP production plant in Shandong Province, China, 2016	129–1442 ng/m^3^ and 91–333 ng/m^3^, respectively
	PM2.5 samples	Jinan, China, 2016	9.80 to 105 (mean: 38.7) ng/m^3^
	Air	Shergyla Mountain and Lhasa on the Tibetan Plateau of China, between 2012 and 2015	130 and 1300 pg/m^3^ and 1100–14,440 pg/m^3^, respectively
	Household air	Norway, 2012	94 to 151 (mean: 128) ng/m^3^
	House dust	Canada, between 2007 and 2010	4.0–57 (mean: 6.2) μg/g
	Household dusts	Bavaria, Germany	4–27 μg/g
	Indoor dusts	Australia, 2015	0.29 to 58 (mean: 9.4) μg/g
	Air	Zurich, Switzerland, 2011 and 2013	1.8 to 17 (median: 4.3) ng/m^3^ and 1.1 to 42 ng/m^3^ (median: 4.7), respectively
	Air	Japan, South Korea and China, 2008	2.26, 2.06 and 137 ng/m^3^, respectively
	Atmosphere	Melbourne, Australia, 2013–14	28.4 ng/m^3^ in summer to 1.8 ng/m^3^ in winter
	Atmosphere	Antarctica, 2012	9.6 to 20.8 (mean: 14.9 pg/m^3^)
Aquatic system	River water	Shanghai, China, 2016	0.278 μg/L
	Influents of wastewater treatment plants	Barcelona, Spain, 2014	0.5 μg/L
	Surface sediments	East China Sea, 2012	5.8 to 64.8 (mean: 25.9) ng/g dw
	Surface sediments and bivalves	Bohai Sea in China, 2014	97.4–1756.7 ng/g dw and 476.4–3269.5 ng/g dw, respectively
	Seawater and sediments	The Pearl River Estuary, Southern China	180 to 460 ng/L and 180 to 620 ng/g dry weight (dw), respectively
Soils	Paddy field soils	An e-waste dismantling area and China between 2008 and 2010	30.4 to 530 (mean: 80.2) ng/g dw
	Paddy soils	Liaohe River Basin China, 2010	61.5–171.1 ng/g dw
	Soils	The Pearl River Delta in South China between 2009 and 2010	1.9 to 236 (mean: 18.3) ng/g dw
	Urban soils	Shanghai, China, 2011	<LOD–615 (mean: 39.4) ng/g dw
	Suburban soils	Shanghai, China, 2011	<LOD to 697 (mean: 18.8) ng/g dw
	Soils	One background area in Shanghai, China, 2011	0.42 to 420 (mean: 9.6) ng/g dw
	In-plant and ambient surface soils	A Chinese CP production plant Dalian, China, between 2013 and 2014	1421.4 and 141.9 ng/g dw, respectively
	Surface soils	Nationwide agricultural lands China, 2016	39 to 1609 ng/g
	Soils	Taizhou, China, 2017	68.5–2.2 105 ng/g dw
	Coastal soils	Shandong Peninsula, China, 2017	50.06–266.3 (mean: 93.97) ng/g dw
	Soils	UK and Norway, 2008	24 ± 72 ng/g soil organic matter
	Soils	Switzerland, 1994 and 2014	35 vs. 3.0 ng/g, respectively
	Soils	Arctic between 2011 and 2012	7.1 ± 0.7 ng/g dw
Biota	Common barbell Barbus	The Rhone River Basin, France, 2009	63–1492 (median: 728) ng/g ww
	Aquatic animals	Bohai Bay in Northern China, 2014 and 2017	86 to 4400 (mean: 940) ng/g ww
	Fish	Liaodong Bay, North China, 2014	376.3 to 8596 (mean: 2131) ng/g lw
	Fish	Alpine lakes and the Lhasa River on the Tibetan Plateau in 2019	26.6 ng/g dw
	Wildlife species	Greenland between 2012 and 2014	Median concentrations of 0.18–2.4 μg/g ww
	Fish, seabirds, marine mammals and terrestrial birds and mammals	Scandinavia between 2006 and 2017	26–1500 ng/g lw
	Aquatic species	Aquatic species	10 to 1300 μg/g lw
	Coniferous plants	A chlorinated paraffin plant in Dalian, China, between 2013 and 2014	1738.7 ng/g dw
	Humpback whales	Antarctica	46 ng g/g lw
Human diets	Cereal legume meat	19 Chinese provinces in 2010	343 and 328 ng/g ww, respectively
	Aquatic foods	18 Chinese provinces in 2011	215 to 4200 (mean: 1472) ng/g ww
	Home-produced eggs	An e-waste site in South China, 2013 and 2016	477 to 111,000 ng/g lw
	Raw food materials	Beijing, China, between 2014 and 2016	0.67 to 5100 ng/g ww
	Diets for the general population	Beijing, China, 2016	24.6–546 (mean: 83) ng/g dw
	Mrigal carp, white amur bream, yellow catfish, pork, chicken, duck and chicken eggs	Qingyuan County, South China, between 2016 and 2017	408, 4.57, 5.64, 49.2, 43.4, 66.1 and 4.84 μg/g lw, respectively
	Composite food	Beijing, China, 1993 and 2009	200 to 600 pg/g and 8500 to 28,000 pg/g, respectively
	Composite food	Japan, 1993 and 2009	<LOD–290 pg/g ww and < LOD–1100 pg/g ww, respectively
	Chicken eggs	An e-waste recycling region in China, 2013	2600–6800 (mean: 4000) ng/g lw
	Vitamin E dietary supplements	German market with data not indicated	<LOD to 61,000 (mean: 3810) ng/g fat
Human tissues	Blood	China in 2012	370–35,000 (median: 3500) ng/g lw
	Maternal and cord serum	Beijing, China, 2013	21.7–373 ng/g ww and 8.51–107 ng/g ww, respectively
	Plasma	Dalian, China, 2015	<LOD to 203 (mean: 32) ng/g ww
	Human milk	Eight Chinese provinces, 2007 and from 16 Chinese provinces, 2011	303 and 360 ng/g lw
	Human milk	12 Chinese provinces in 2007 and 16 provinces, 2011	681 and 733 ng/g lw, respectively
	Human milk	China between 2014 and 2015	2.51 μg/g lw
	Human milk	UK between 2001 and 2002	0.18 μg/g lw
	Placenta	China, 2018	98.5–3771 ng/lw

Data were compiled from a table by Wang et al. (2019b)

## Research gap and priorities on SCCP studies in Africa

Africa is the second largest continent in the world covering over 30 million km^2^, including over 54 countries and a population of about 1.17 billion people (UNDP 2015). Apart from the three SCCP studies conducted in Africa (Adu-Kumi-Jonathan et al. 2019, Brits et al. 2020, Möckel et al. 2020), it remains the least studied continent in the world with respect to SCCP studies in different matrices. However, about 199 CP studies have been published in other continents in matrices such as river, sediments, soil, fish, human blood and serum, consumer products and landfill sites, which reveals that there remains a huge research gap in the continent of Africa. Studies have further indicated that the analysis of SCCPs is a challenging and demanding task due to the complexity of their industrial mixtures and interferences with other organic compounds; therefore, only a few laboratories are involved in their quantification (van Mourik et al. 2015; Schinkel et al. 2018). Considering that Africa is a developing continent, factors such as lack of technical support may be some of the reasons behind the limited conducted studies on SCCPs. Currently, there are no published reports on SCCP concentration in consumer products, landfill leachate and sediment samples in Africa. Furthermore, though the production and use of SCCPs have been prohibited in some countries including the US, Japan and Brazil because of their toxicity and effects in the environment, there are no immediate actions or regulations on the production and use of SCCPs in Africa. The current climate change, which is being felt across Africa and the rest of the world, will exacerbate the situation if no measures are taken to ameliorate the situation. Furthermore, information about environmental levels of SCCPs in Africa is essential for guiding efforts to reduce health implications associated with the contaminant in order to achieve the United Nations’ Sustainable Development Goals (SDGs) on healthy lives and the promotion of well-being for all at all ages by 2030. There is, therefore, a critical need to study environmental concentrations of SCCPs in Africa as well as to expand sampling sites to include landfill leachates, consumer goods and other matrices. Historical sources, current sources and Africa’s characteristics make Africa an important study area. In addition, Africa needs to assess the presence, use and life cycle of SCCPs in the continent and develop a National Implementation Plan with specific activities. Moreover, there is a need of developing SCCP inventories of which the Stockholm Convention has already developed a guidance document in this respect (UNEP 2019a).

## Concluding remarks

In comparing SCCP studies done in Africa so far with those published in other continents, it is clear that there remains a huge gap in CP studies. There are limited published studies on the determination of SCCP concentration in consumer products and landfills as well as in other different matrices in the continent, and as such, further research studies are necessary in order to tackle those research gaps. Additionally, very few studies have so far reported on SCCP concentration in landfill leachate and sediment globally. Nearly all imports of CPs and CP-containing waste end up in dump sites or landfills in Africa. The lack of resources on intensive analytical methods and elaborate sampling techniques required may be the reason for fewer SCCP studies in Africa; therefore, attention should be given to this area. Temperature is one of the key meteorological parameters that can severely influence the global distribution of SCCPs in the environment. Due to the hot climate experienced in Africa, SCCPs can be evaporated and thereby influence the re-emission and distribution of SCCPs to other regions. In view of the effects of SCCPs globally, the use of SCCPs needs to be legally restricted/banned and not used in many exempted applications as prescribed by the Stockholm Convention. Robust SCCP inventories and other CPs containing SCCPs are overdue in Africa and, therefore, require some attentions. It is, therefore, important to gather comparable data on concentrations of SCCPs in consumer products and other matrices in all the continents in order to have a global overview of the state SCCPs. It is also crucial to study SCCPs in Africa in order to understand the impacts of the Stockholm Convention to reduce POPs in Africa and to assess their distribution and influence of other continents to the environmental burden of SCCPs in Africa, and vice versa.

## Data Availability

Not applicable
